# Women and agricultural productivity: Reframing the Issues

**DOI:** 10.1111/dpr.12243

**Published:** 2017-06-26

**Authors:** Cheryl R. Doss

**Affiliations:** ^1^ Department of International Development Oxford University

**Keywords:** agricultural policy, agricultural productivity, developing countries, gender, smallholder farming

## Abstract

Should agricultural development programmes target women in order to increase productivity? This article analyzes the challenges in distinguishing women's agricultural productivity from that of men. Most of the literature compares productivity on plots managed by women with those managed by men, ignoring the majority of agricultural households in which men and women are both involved in management and production. The empirical studies which have been carried out provide scant evidence for where the returns to projects may be highest, in terms of who to target. Yet, programmes that do not consider gendered responsibilities, resources and constraints, are unlikely to succeed, either in terms of increasing productivity or benefitting men and women smallholder farmers.

## 1 INTRODUCTION

To what extent should agricultural development investments explicitly target women? Is it possible to use investment in agricultural technology to redress gender imbalances and to reduce poverty? Would a women‐focused agricultural investment strategy yield a double dividend—increasing gender equity at the same time as increasing overall productivity? These and other questions have become pressing issues for development policy and strategies.

The development literature abounds with claims about the benefits of targeting agricultural investments at women, especially in sub‐Saharan Africa. These claims take many forms, but in general it is argued that increasing women's agricultural productivity is key to increasing overall agricultural productivity, empowering women and reducing poverty.

In general, the arguments for targeting women can be grouped into two main strands. One strand focuses on the productive potential of women farmers. The claim is that women are heavily involved in agricultural production in the developing world—and especially in Africa—and that they have been left out of many development efforts. Thus, there are very high returns to targeting current investments to women—with these returns showing up as increased aggregate agricultural production and higher incomes for women.

A second (and not mutually exclusive) line of argument posits that women represent an important class of beneficiaries of agricultural development efforts—and that their needs have frequently been neglected by programmes that focus on productivity increases. Because many poor women are farmers, and many poor farmers are women, there are reasons to direct agricultural development towards this group. The importance of women as beneficiaries is increased by the instrumental roles of women with respect to child health, nutrition and education. Improving the well‐being of women and offering them expanded opportunities will both increase their own welfare and have the potential to create positive effects on the next generation.

Both of these strands of argument incorporate a range of hypotheses, and both rest on plausible and internally consistent theories of change. In essence, they are arguments that the social rates of return on agricultural development investments are higher when those investments are targeted at women.

The empirical basis for these arguments is not as well developed, however. Evidence is limited as to the social benefits of targeting research or other agricultural interventions at women. The claim of efficacy relies on several steps in a complex causal chain: first, that investments can selectively drive up women's productivity; second, that these increases in women's productivity will succeed in producing benefits for women; and third, that the resulting social rates of return for these investments are higher than for other development investments.

Many voices in the development community take it as given that these causal links are valid, and donors increasingly require that gender issues be addressed in projects and proposals. Other voices continue to express scepticism about a women‐focused strategy in agriculture—or at least suggest that there may be trade‐offs associated with targeting interventions at women. However, empirical analysis of these issues has been sparse.

New data has allowed us to challenge some myths about women in agriculture. Evidence for Africa (Doss, Kovarik, Peterman, Quisumbing, & van den Bold, 2015) and Asia (Kieran, Sproule, Doss, Quisumbing, & Kim, 2016) is inconsistent with a widely cited figure that women own only between 1% and 2% of the world's land, although they do own considerably less land than men. In the six African countries for which there is data, women provide 40% of the labour for crop agriculture, a lower figure than the 60% to 80% that is often cited (Palacios‐Lopez, Christiaensen, & Kilic, 2017). Finally, no evidence supports the claim that women produce 60% to 80% of the world's food (Doss, 2014). Given women's reponsibilities for household work, it would be surprising if they produced most of the food.

Careful measurement has also cast doubt on earlier claims about gender gaps in agricultural producitivty. An initial review of the early literature (Quisumbing, 1996) found that while women have lower output on their plots of land, this could be attributed to their having lower quality land, less access to fertilizer and other inputs, and less credit and extension support inputs. Following a more recent review, the FAO State of Food and Agriculture Report in 2010‐11 (FAO, 2011, p. 5) took this further: ‘If women had the same access to productive resources as men, they could increase yields on their farms by 20–30 percent. This could raise total agricultural output in developing countries by 2.5–4 percent, which could in turn reduce the number of hungry people in the world by 12–17 percent.’ These are plausible esitimates, but in this era when the gold standard of evidence is randomized control trials, note that these numbers are calculated using the estimated production functions for women assuming that they used the same levels of inputs as the men. They are not the result of programmes which provide men and women with equal levels of inputs.

Beyond the statistics, some of the discussion around women‐focused agricultural development involves rather blurry conceptual thinking, both from advocates and sceptics. As this article will argue, it is not clear what is meant by ‘women's productivity’. This is a difficult concept to define, let alone to measure. What would it actually mean to identify food as being ‘produced by men’ or ‘produced by women’? Are these useful ways to think about the gendered structure of agriculture and food systems? Across the world, most farms are operated by families that include both men and women, so how can we disentangle their individual contributions?

Much of the academic literature on women's agricultural productivity has gone in directions that are not helpful in shedding light on the central policy questions. Similarly, the policy debate has often highlighted the wrong issues. The literature offers little insight into the key question—which is, simply put, where are the returns to development investments highest? Nor does it provide much insight into the narrower questions of how to increase agricultural productivity and ensure that women are able to benefit from these gains.

Yet, regardless of how investment priorities are set, gender is an essential analytic category for understanding the impacts of agricultural development investments. This is true whether or not women are targeted as the users of technology or as beneficiaries. Farming and food preparation are deeply gendered activities. The impacts of agricultural technologies and development interventions are necessarily filtered through the gendered patterns of agricultural labour, household enterprises, family food consumption decisions and social structures. Agricultural technologies are not, in general, gender neutral. Thus, a gender lens is essential for assessing the effectiveness and impact (whether ex ante or ex post) of an agricultural technology or intervention.

The article begins by considering the conceptual and methodological challenges to separating men's and women's agricultural productivity. The empirical literature is briefly reviewed, highlighting the main methodological approaches and their contributions. None of the literature adequately addresses the issue of separating women's production in households with joint production, even though these account for most of the world's farm households. The final section discusses new potential directions for research on women's agricultural productivity.

## 2 CONCEPTUALIZING AND MEASURING WOMEN'S AGRICULTURAL PRODUCTIVITY

In considering how to increase women's agricultural productivity, the first challenges are to conceptualize and measure it. Each of the approaches used has advantages and disadvantages and different data requirements. None is well suited to measuring separately the productivity of men and women, so additional assumptions are needed to arrive at sex‐disaggregated measures. The additional assumptions in turn have the potential to affect the conclusions concerning women's productivity levels—both in absolute terms and relative to men.

Productivity reflects a relationship between inputs and outputs. The two most widely used measures are partial productivity measures—output per unit of a single input. Land productivity is defined as output per unit of land; labour productivity is output per unit of labour effort. In both cases, output may be defined in either physical quantities (such as kilograms of grain) or economic value. Labour input is normally defined in terms of hours or days, but in some cases it is measured in value terms—for example, when hired labour is aggregated with family labour.

### 2.1 Conceptual issues regarding agricultural productivity by sex

To analyze women's agricultural productivity, one approach that has been widely used is to consider the ‘household farm enterprise’ as the production unit and to compare the productivity of different households, distinguishing between male‐ and female‐headed households. While this approach is relatively simple, it ignores the contributions that women make to farms in households headed by men (and conversely the contributions that men make to farms in households headed by women). This not only ignores the joint structure of most households, it also introduces a possibly spurious comparison between households that differ in many other ways. Cultural norms frequently dictate that a household will not be defined as female‐headed if there is an adult male present. Thus, female‐headed households almost invariably have fewer adult members (and no male members). A comparison of female‐headed households and male‐headed households tells us almost nothing about men and women farmers; it primarily tells us about the influence of household structure.^1^

A second approach is to consider the sex of the farmer of a particular plot or set of plots. This approach allows for multiple farmers to be identified in a given household. Typically, output from a plot is assigned to an individual farmer, defined either as the person who ‘manages’ the plot or ‘controls output’ from the plot.

This plot‐level approach has largely replaced a third option—widely used in older studies with more limited data—namely, to assign the output of particular crops to different household members. For example, if cultural norms suggest that cocoa is a man's crop and millet is a woman's crop, the household output of these crops would be assigned on this basis. This approach is particularly problematic if we are trying to follow changes over time, since the patterns of who grows what is likely to change.

This discussion raises the question of how a farmer is defined. A farmer may be defined as the owner, the manager or the person providing the day‐to‐day labour for the plot or crop. Because women are less likely to have land rights, they may not be considered to be farmers. In some cultural contexts, women may be seen as farm helpers, but not farmers; or they may be viewed as only growing food in the kitchen garden or homestead (Twyman, García, & Muriel, 2015). If we misidentify farmers, then our measures of men's and women's productivity will not be useful.

Plot‐based and crop‐based measures of output almost always omit activities related to livestock. This is potentially a very important omission: livestock‐related activities account for a large fraction of total agricultural production in almost all farming systems—and often for even larger fractions of cash income. For example, FAO (2009) reports that livestock contribute 40% of the global value of agriculture output. Animal care is often highly gendered—with women responsible for dairying and smallstock in many societies, while men are typically responsible for ranching and herding. Thus, the omission of animal agriculture may have important implications for the measurement of men's and women's relative agricultural productivity.

The remainder of this section focuses on the challenges in obtaining productivity measures. These are difficult to compute in any data, and the problems are exacerbated when we try to use these approaches to separate out women's productivity.

### 2.2 Measuring productivity: Land

The most common measure of agricultural productivity is crop yield—the physical quantity of output per unit of land, for a single crop. This approach works best when farmers plant one crop on a plot of land. The measurement itself is not trivial; accepted practice is to follow complex protocols for conducting ‘crop cuts’ at harvest time, with concomitant protocols governing technical issues, such as the moisture content of the crop at the time that the output is weighed. Yield may also be estimated through farmer recall of how much was produced, along with measures of plot area.^2^

Yield measurements are less straightforward in areas where there is multiple cropping per year on a single plot of land, or where several crops are simultaneously planted. The picture becomes even more blurred when different crops are grown in different seasons, or when multiple crops are cultivated simultaneously on the same field, as is common in many African production systems. Even harder to measure are yields of crops that are continuously harvested (e.g., vegetables) or that are harvested piecemeal (e.g., cassava).

When we are interested in the productivity of multiple crops, there are three possible approaches. The simplest, used often with yield, is to allocate the input of land or labour to each type of output. For example, if an acre plot is intercropped equally with maize and beans, then half an acre would be considered to be planted with maize and half an acre with beans, and the yields calculated accordingly. A second option is to treat intercropped plots as though they contained separate crops for the purposes of analysis. Thus, we might analyze plots planted to maize, those planted to beans, and those intercropped with maize and beans.

The third option is to aggregate across crops using value of output, usually in farm gate prices. This approach is conceptually very clear and has the advantage of allowing comparisons across crops and cropping systems. However, a given quantity of physical output may receive different prices in different years (or even on different days), depending on total supply and the availability of local markets. And men and women may report or obtain different prices for the same produce.

Large‐scale surveys typically find large differences in yield across plots of land, even within geographically narrow areas. There may be differences in plot‐level characteristics, such as the soil quality or availability of irrigation. Shocks, such as weather or exposure to pests or disease, may vary. Plots may have different access to roads and markets. Other differences may result from the choices of farmers, such as choices of crop or variety, use of fertilizer, and particular management practices. Finally, farmer characteristics may be important, such as farming experience or level of education. The sex of the farmer may be correlated with all, some or none of these factors.

This means that comparisons of yield for men and women farmers are difficult both to make and to interpret even when we can assign the output to an individual farmer. Where men and women own and/or manage separate plots within a household, the yield differences could be due to the managerial abilities of the men and women farmers, or to their physical strength or other attributes. But they may also be due to differences in the plots or to choices that households make about how to allocate resources and prioritize investments across a range of agricultural and non‐agricultural activities. Thus, a comparison of yields on male‐ and female‐owned or managed plots has little or nothing to tell us about the potential returns to investments in agriculture that target women.

### 2.3 Measuring productivity: Labour

Labour productivity measures the output per unit of labour. Measuring labour inputs presents challenges, even before we begin to disaggregate by gender. Labour is often measured in days worked; farmers are asked to estimate how many days were spent working on each task. Often farmers are asked to recall this for the previous farming season. It implicitly assumes that a day's work is a useful measure of input and that the contribution of each day's work is roughly equal. These measures rarely account for hours worked or effort expended.

A day of labour will have a different marginal contribution to output, depending on the age, sex, health status, etc. of the worker. Some workers will put in longer days and more effort. For a variety of reasons, including social norms, skills and physical capabilities, there may be differences in the labour provided by men and women. In addition, because women typically have much higher burdens in terms of the time and physical requirements of providing care labour—such as caring for children, preparing meals, fetching water and doing laundry—they have less time per day available for agricultural work. In addition, the returns to a day of labour will vary across the agricultural season and activity; the contribution of a day spent weeding may be different from the contribution of a day spent clearing the field. The opportunity costs of labour for men and women may also differ; non‐farm opportunites for earning income may be lower for women.

The usual measure of labour input—time spent working on a farm or plot—does not necessarily account for knowledge or management skills. A family member might work few hours, but provide knowledgable direction to others; this will not be well accounted for in the data and the results may be less meaningful. Labour provided by women and men may be complements, rather than substitutes (as assumed implicitly in much of the literature).

When calculating labour productivity, output measures are often calculated in terms of value, so that they can incorporate a range of crops. The variability of agricultural production across seasons generates a lot of statistical ‘noise’ in production data, which means that labour productivity is not calculated with much accuracy in any season. To distinguish differences in labour productivity between two farms or two individuals, we would typically need lots of observations to extract ‘signal’ from noise—even if everything else were equal.

Numerous studies have tried to estimate labour productivity for men and women separately, and new household survey data such as the World Bank's Living Standards Measurement Study ‐ Integrated Surveys on Agriculture (LSMS‐ISA) provide fairly detailed estimates of labour use on each plot by different individuals.^3^ But it is difficult to extract meaningful measures of individual productivity from these data, and even then it is hard to know how to interpret measures of labour time. Much agricultural labour is carried out jointly with other household activities. How should we count hours that a woman spends working on the farm while supervising or caring for her children? Or the time that she spends tending backyard livestock while carrying out other activities around the homestead? Women's measured productivity may be lower if we are only counting some of her outputs. Indeed, how should we account for cultural views of labour that may not consider some women's tasks as ‘work’? Studies of labour productivity typically gloss over these problems, and comparisons of men's and women's labour productivity are consequently problematic.

### 2.4 Measuring profitability

Economic theory suggests that farmers will seek to maximize the profitability of their farms, rather than maximizing the output of one or more crops. Farms may include both crops and livestock, as well as other income generating activities. Thus, yield and labour productivity can give a misleading impression by only considering one of the farm activities. In addition, increasing yield may actually decrease profits; such as when fertilizer and improved seeds are expensive relative to farm gate prices of outputs.

Estimating profitability requires information on the monetary cost of both outputs and inputs. Value added measures are calculated by subtracting the cost of purchased inputs, such as fertilizer and seeds, from the value of output.^4^ Prices may vary from one farm to the next, due to ‘last mile’ transport costs and quality differences. Prices may also vary across the season. The costs of factors of production (typically, land, labour and capital) are not included in these measures of agricultural profitability. Identifying the costs of these factors of production can be challenging, especially when the markets for them are thin. In addition, the opportunity cost of labour varies by individual and across the season.

For this reason, economists prefer measures of productivity that account for many different inputs and factors of production, such as ‘multi‐factor productivity’ or ‘total factor productivity’ (TFP) measures. TFP measures compare aggregate outputs to aggregate inputs. Calculating TFP raises challenges of its own and requires a number of assumptions. Unlike yield differences across plots, a measure of TFP would at least address the problem that some of those differences emanate from differential input use and differences in quantities of labour and capital.

As with measures of yield and labour productivity, TFP exhibits wide heterogeneity across plots, due to factors such as localized weather. Calculations are also quite sensitive to assumptions (such as the form of the production function). Often TFP is used to analyze changes over time. We say that TFP has increased when the index of outputs increases faster than the index of inputs. Yet, for differences in TFP to be convincing, we need many data points, so that we can be sure that the variations in output due to weather and other short‐term factors are not the driving factors. This might require multiple observations over time, or lots of observations of the same farmers under different circumstances. Comparing profitability or TFP for men and women farmers is thus very demanding in terms of data. These comparisons assume that inputs and outputs are measured well. It is also essential that inputs and outputs are measured equivalently for men and women, so that (for instance) women's labour time is not overstated when they are engaged in other activities concurrent with farming.

### 2.5 Crop choice and input use

Two other issues are particularly relevant for considering gender gaps in agricultural productivity: the choices of crops and inputs. Productivity measures usually take crop choice as given and ask, for example, whether men and women are equally productive as maize farmers. If households decided randomly which individuals cultivate which crop on which plot, then these comparisons would be straightforward. In reality, however, this tends not to be the case. If households systematically assign the best plots to men, and if these plots are used to grow maize primarily for the market; whereas the worst plots are assigned to women to grow millet intercropped with beans for home consumption, how do we make comparisons? What would it mean to claim that men are more productive at growing maize than women are at growing millet or beans? Men and women farmers face different access to inputs, information and markets, and thus they will make different choices about what to grow. These choices may be made jointly, as part of a household strategy, or they may be made separately with little discussion or co‐operation. Decisions as to what to grow (and how to grow it) may reflect profit‐maximizing strategies or they may be driven by social and cultural norms.

The question of crop choice is also related to the extent to which men and women are producing for the market or home consumption. When producing partly or entirely for home consumption, farmers may pursue a range of complex objectives. When there is limited access to markets, with a high wedge between the prices farmers receive as producers and the amount that they pay as consumers, it can be economically rational for them to produce lower‐value crops for home consumption rather than more valuable crops for the market.

In addition, men and women may have different access to markets to sell their produce. They may value different traits for the same crop (e.g., taste and cooking characteristics for home‐consumed crops, versus yield or cost advantage for marketed crops). Men and women may have different access to tools and machines, and this may affect both their choices of crops and the techniques of cultivation.

Input use is also endogenous. To pursue further the example above, men and women growing maize on different plots may rationally choose different types and quantities of inputs. A number of factors will affect input use, including prices, transport costs, or the units in which inputs are available for purchase. Gender conditions all these factors. Men and women may also have different access to information about technologies and markets, both through extension services and through their own informal networks.

Two analyses consider the actual provision of inputs or input subsidies in Malawi. Karamba and Winters (2015) find that the provision of fertilizer subsidies at the household level increases productivity on plots managed by men and those managed by women. But the increases in women's productivity are not disproportionately large enough to reduce the gender gap in productivity. An earlier study found that when men and women farmers were provided with inputs as part of a national trial, there was no gender gap in productivity (Gilbert, Sakala, Webster, & Benson, 2002).

While identifying productivity differences between husbands and wives does not necessarily mean that the household as a whole is not optimizing, economic efficiency conditions would normally imply that the marginal product of all the inputs, including labour, would be the same across plots. If this is not the case, then it suggests that there are some missing markets within households. A variety of reasons may limit the allocation of resources between spouses, including information failures, power dynamics or social norms.

There is a range of other ways in which gender may influence the choices that farmers make about what to grow and how to grow it. In general, women have smaller—and often poorer quality—plots of land. Thus, the scale of farming may differ between men and women. Access to markets may differ by gender; some markets are not considered appropriate places for women, and access to transportation may vary. Some evidence suggests that there may be systematic gender differences in the prices received by men and women for the same output. Women may sell more of their produce at the farm gate, if their time is limited by household responsibilities that reduce their ability to travel to the market or because they are selling smaller quantities and it is not worthwhile to make the trip to the market. Social strictures may make it more difficult for women to bargain effectively with male traders for higher prices. Certainly, not all of these factors are present in every community; some women bargain well in the market and travel long distances to sell their produce. But it is important to consider the ways in which gender may mediate many of these market activities and thus choices regarding agricultural production.

## 3 EMPIRICAL ESTIMATES OF GENDER DIFFERENCES IN AGRICULTURAL PRODUCTIVITY

In spite of these challenges, there is a large and growing empirical literature that claims to estimate the gender differences in agricultural productivity. One motivation for much of this literature is to ask whether women are inherently less productive farmers (or perhaps more productive farmers), or whether they simply face a different range of constraints than those facing men.

A range of empirical approaches have been used to estimate the gender differences in agricultural productivity. To some extent, these different approaches are asking different questions and, thus, it is useful to consider this literature by the approach that is used.

### 3.1 Estimating production and profit functions

Regression analysis can estimate the contribution of various inputs to output, whether measured as yield or value. Researchers may use a right‐hand side dummy for the sex of the plot manager—or perhaps include men's labour and women's labour separately. The intuition is that these regressions establish whether there is a significant relationship between the sex dummy variable and yield or profit. In general, this literature finds little or no significant difference in men's and women's agricultural productivity after controlling for access to inputs, plot, farmer, and household characteristics (Moock, 1976; Adeleke, Adesiyan, Olaniyi, Adelalu, & Matanmi, 2008; Saito, Mekonnen, & Spurling, 1994; Hill & Vigneri, 2011). This is usually interpreted to mean that women would be as productive as men if they had equivalent opportunities.

Most of these analyses compare the productivity differences of men and women across the entire sample. In contrast, Udry (1996) considers productivity differences among men and women within the same households in Burkina Faso. He finds large intra‐household differences in productivity across men's and women's plots and attributes them to differences in the intensity of the application of labour and fertilizer. He estimates that shifting labour and fertilizer from men's plots to women's plots within the same household would substantially increase total household output. This is one of the few studies that considers the impact of reallocating inputs, rather than providing women with an amount similar to that of men.

Some of the more recent literature wrestles with the underlying methodological issues originally identified by Quisumbing (1996). Many of the studies above only consider one crop; one reason that women obtain lower values for their agricultural production overall may be due to the mix of crops that they produce. The analyses that consider crop choice find that even if men and women are equally productive in farming a given crop, women systematically produce crops of lower value and, thus, their overall profitability is lower (wa Githinji, Konstantinidis, & Barenberg, 2014; Peterman, Quisumbing, Behrman, & Nkonya, 2011).

### 3.2 Estimating labour productivity

A related set of studies compares the labour productivity of men and women in agriculture. The advantage of this approach is that it does not require that output be allocated to individuals. Instead, it estimates how the labour inputs of men and women affect total farm productivity. The challenges of measuring labour inputs are discussed above.

The usual approach is to treat men's labour and women's labour as separate inputs in an agricultural production function. Household output value (or some other measure of production) is regressed on a set of inputs, including men's labour and women's labour, and the elasticities are compared. Because results can be quite sensitive to the specification of the functional form for the production function, it is desirable to use a relatively flexible functional form.

Some studies find no gender gap in labour productivity (Aly & Shields, 2010). Others find that the patterns vary across seasons (Kumar & Hotchkiss, 1988), or that the marginal product of female labour is lower than that of male labour (Jacoby, 1992; Laufer, 1985).

Because men and women typically specialize fully or partly in different types of farm labour, it is difficult to interpret these findings. If we know that, on the margin, an hour of women's time spent weeding contributes less to overall productivity that an additional hour of men's time spent in preparing the land, how would this change the types of interventions that are proposed? If the marginal product of women's labour is high, this could imply that it would be useful to free up some additional time for women to contribute to agriculture. But it is equally true that if the marginal product of women's labour turns out to be low, it might support an argument that there are particularly high payoffs to redressing this imbalance.

### 3.3 Stochastic frontier analysis

Stochastic frontier analysis is a non‐parametric technique that estimates the efficiency of individual production units relative to a frontier. These efficiency measures can then be regressed on a variety of explanatory variables to see how much of the efficiency differences can be accounted for by different variables.

In this approach, output is regressed on factors of production and then the estimates of inefficiency/error term are regressed on factors (e.g., farmers’ characteristics, plot and household characteristics) hypothesized to result in output being lower than the maximum. This approach is widely used in the agricultural economics literature, but has only been used to a limited extent to consider whether inefficiency is correlated with the sex of the farmer. Two studies find that women and men have similar technical efficiency (Kinkingninhoun‐Mêdagbé, Diagne, Simtowe, Agboh‐Noameshie, & Adégbola, 2010; Overfield & Fleming, 2001) In Nigeria, among cassava farmers, Timothy and Adeoti (2006) find that women have higher technical efficiency, but men have higher allocative efficiency.

Again, this literature can be helpful in identifying the constraints that farmers face in attaining higher levels of efficiency and how they differ for men and women. But it does not provide clear guidance on how to effect these changes and what types of interventions would have the highest returns. Like TFP analysis, it depends critically on perfect measurement of all inputs and outputs.

### 3.4 Decomposition of productivity differentials

Decomposition analyses allow us to distinguish between (1) the quantity and characteristics of land, labour and other inputs controlled by women, and (2) the structural effects, which are gender differences in the returns to these factors.

A recent set of papers use the LSMS‐ISA data and Oaxaca‐Blinder decomposition analysis to identify these patterns in six countries. These results are summarized in World Bank & ONE Campaign (2014). The value of total crop output per hectare is compared across plots managed by men and women. Analyses from four countries and southern Nigeria (Ethiopia, Malawi, Niger, southern Nigeria and Uganda) find gender gaps in productivity when simply comparing the differences in value of output. Controlling for plot size and geographic factors, the gender differences generally increase rather than decrease. In Niger, the gender gap increases from 19% to 66%. While it disappears in Nigeria (South), a gender gap of 46% results in Nigeria (North) and a gap of 33% in Uganda.

One of the reasons for these dramatic differences is that women, on average, have smaller holdings than men. For Malawi, Niger, Nigeria (North and South) and Uganda, after accounting for the differences in farm size, the gender gap widens. In these countries, among smallholder farms, there is an inverse relationship between farm size and productivity; smaller farmers are more productive per unit of land. Using this information to simulate what would happen if women had larger farm sizes, similar to those of men, they find that women would be even less productive. This finding has the perverse implication that the productivity of land could be increased by dividing farms and making them smaller. It underscores the fact that a focus on land productivity may be misplaced; we may care much more about the productivity of labour, which is more closely linked to the well‐being of the poor.

The main objective of these studies is to move beyond quantifying the gender gap to identify the contribution of each factor to the gender gap in productivity. This provides more information on the patterns, but again does not tell us which types of interventions would have the best impact.

### 3.5 Technology adoption

When detailed production data are not available, technology adoption is often used as a proxy for increased agricultural productivity. The implicit assumption is that farmers will only adopt improved technologies if they increase productivity or, more accurately, if they increase profit or well‐being.

This approach does not require detailed information on the use of inputs and outputs. In particular, it does not require that the researchers calculate a value for labour inputs; instead, we simply assume that farmers take labour requirements into consideration when deciding whether or not to adopt.

An extensive literature attempts to identify which farmers use improved technologies. In their simplest form, these studies use regression analyses to identify the characteristics of adopters and include a variable for the sex of the farmer. A number of studies suggest that, all else being equal, men and women are equally likely to adopt these technologies. The challenge is that rarely is all else equal. In addition, simply knowing which farmers are using improved technologies does not tell us which policies will increase adoption.^5^

What can we learn from looking at technology adoption? It is useful to know the extent to which men and women are using various technologies, and the extent to which differential technology adoption can be explained by access to land, information and other relevant inputs. But even if we find that there are unexplained gender differences in technology adoption, this does not tell us whether there are potentially new technologies that women farmers would adopt to increase their productivity.

### 3.6 SUMMARY

A large literature has sought to compare the productivity of men and women in agricultural activities in developing countries. This proves to be a challenging task, starting at a conceptual level and continuing to measurement, estimation and interpretation. It is not always clear what question is addressed by this literature. What could we hope to learn from this literature, even if all the conceptual and measurement issues could be solved and the endogeneity problems resolved? If we find that women have lower measured productivity than men, what can we conclude about the potential returns to investments that target women? If we want to increase aggregate productivity, should we invest less in women, since they are less productive? Or more, since there would seem to be easier opportunities to increase their productivity with off‐the‐shelf technologies and interventions? If we find that productivity differs, but only because of inputs, what is an appropriate response? The literature has little to say on these questions; even the most powerful econometric tools do not allow us to answer the key policy questions.

## 4 MEASURING WOMEN'S PRODUCTIVITY WITHIN JOINT PRODUCTION SYSTEMS

A further difficulty arises in estimating women's productivity in agriculture because most farming takes place in households in which men and women share responsibility for the same plots and for the whole of the household enterprise. Other than the analyses of labour productivity, most of the literature compares productivity on men's and women's plots. There is often a gender division of labour in these household enterprises, with men and women focusing on different agricultural tasks, including management decisions; nevertheless, both men and women are involved in much of the agricultural production, and their contributions are not easily separated.

It is not a coincidence that all of the studies comparing productivity on men's and women's plots were carried out in Africa. It is much less common in Asia and Latin America for women to manage separate plots. Even when women do manage distinct plots, these are often viewed as kitchen gardens or homestead plots, rather than agricultural plots. And even in some places in Africa, most of the ‘women's plots’ are managed by women who are the sole heads of their households.

Thus, if we want to consider men's and women's contributions to agricultural productivity—and their potential contributions to agricultural productivity—we must find ways to consider women's contributions to production activities that are jointly—or perhaps entirely—managed by men.

The studies focusing on the marginal product of men's and women's labour provide some information about whether labour is being allocated efficiently within a household, especially when the labour is not perfectly substitutable. Yet, these studies do not tell us anything about women's contributions to farm management or decision‐making. In joint farming systems, both men and women may be involved in management; the degree of involvement may differ and they may be responsible for different sets of decisions.

One option would be to do a much better job of identifying who is involved with each plot of land and find ways to characterize farm labour that incorporates a broader range of management approaches, rather than simply identifying the sex of the manager. This is relevant if plot‐level data are being collected. A useful beginning point would be to distinguish the productivity on plots that were jointly managed from those that were individually managed. Several recent studies have taken a more nuanced approach and considered the productivity of three types of plots: those managed individually by men and women and those managed jointly (Guirkinger, Platteau, & Goetghebuer, 2015; Kazianga & Wahhaj, 2013; de la O Campos, Covarrubias, & Patron, 2016). While the patterns of productivity differ across the locations, these studies caution us against simply dividing plots into men's and women's and suggest that we need to consider joint plots and other management arrangements.

In principle, one way to test these questions would be to design a randomized controlled trial, introducing a new agricultural technology and then randomly providing it to men and women farmers separately and in settings where they farm jointly. An interesting outcome would be to see the differential ways in which the intervention alters household allocations depending on how the technology is targeted. But such an experiment would simply ask, for a given technology, whether it matters how it is distributed. It cannot address the bigger question of which interventions, targeted at whom, will have the highest social returns.

The key issue for policy is whether it makes sense to target investments to women on their own, or women within joint systems, or perhaps not to target women at all. Yet this question is not answered by analyzing gendered patterns of productivity. The reason to target women is not necessarily because they are farming their own plots, but because they are deeply involved in providing labour and management to activities within the broader household production system. Numerous examples suggest that technologies are not widely adopted when they require women's labour but where women receive few or none of the benefits. And some evidence suggests that providing women with information or training may increase household production, even when men are considered the primary farmer or manager.^6^

Thus, there are two issues—how does targeting women and providing them with additional resources, technologies or information affect the total productivity of the household? And how do we ensure that women benefit from the additional output and are thus willing to contribute? This second issue has serious implications for program design. It may involve including payments to multiple people within the same household in commercialization schemes or designing contracts so that women's roles and contributions are visible and compensated.

Simply knowing who contributes labour at the initial stage is useful when designing interventions, but is insufficient for an understanding of existing gender relations. Quantitative descriptions of the baseline situation will not necessarily tell us where the potential exists for transforming gender relations and increasing productivity so that everyone benefits. Nor will this approach identify the potential risks that may worsen women's welfare. Combining the quantitative data with qualitative analysis may provide better insights into the potential processes for change.

The literature on joint production raises some interesting possible directions for policy—or at least for further research. For example, there is consensus that secure property rights promote investment in agriculture. In one of the few analyses that consider gender, Goldstein and Udry (2008) examine land rights in Ghana and find that women invest less in their plots due to their weaker land tenure rights, resulting from their lower political power within the community. But no research has specifically considered the extent to which women may be deterred from investing in agriculture because they may lose access to their land if their husband dies or divorces them. The emerging literature on joint titling of land may be able to provide some insights into this issue.

## 5 CONCLUSIONS

What do we know about women and agricultural productivity in developing countries? How have new data sources shaped and improved our understanding? Will interventions that specifically target women lead to improvements in aggregate agricultural productivity? Will female‐focused agricultural interventions generate higher rates of return than interventions that are, in some sense, gender‐blind?

The emergence of new data sources has allowed for a greatly enriched understanding of gendered patterns of technology adoption, labour use and productivity in agriculture. New data has rejected some of the groundless and unrealistic claims that were made in the past about the share of women's labour in agriculture, or the share of land owned by women.

At the same time, the data show that women are deeply involved in all phases of agricultural production. Their labour at the plot level accounts for about 40% of the total field work in crop agriculture in Africa (Palacios‐Lopez et al., 2017)—and we still lack data on women's participation in other areas of farming, including seed selection and management, input purchasing, output marketing, processing and animal care, not to mention food preparation and cooking. There is widespread consensus that women devote more time than men to many of these activities, and certainly spend more time than men on household labour. As economies continue to grow and transform, we are also reminded that agriculture is only one sector. In some settings, men may be moving out of agricultural work into other sectors that provide better opportunities. This may leave women with greater responsibility for agricultural production, or it may alter the basis on which joint activities are organized.

The new data have allowed for further and methodologically more sophisticated analyses of the relative productivity of men and women. These analyses face a host of conceptual and methodological challenges, but most studies suggest that women would be about as productive as men as farmers, given access to similar resources. Some studies have been interpreted to suggest that there is a potential gain in overall productivity by targeting women, but this evidence is not clear, and there are also studies that suggest the opposite. As data becomes more widely available, it may be possible to identify the conditions under which women have higher productivity and which factors systematically constrain women's output.

Some subjects continue to be poorly addressed in survey data, such as women's land tenure security within households and communities; especially women's ability to retain land in the event of divorce or the death of a spouse. The literature also draws heavily from research from Africa, comparing plots managed by men with those managed by women. The rest of the world may look very different. In particular, in much of South Asia the poor are landless, and the challenges for women in this context may be very different.

Nevertheless, the emerging data can provide important baseline information as we design projects and interventions. They reveal the strongly gendered patterns of labour use and household responsibilities, and in this way they can help identify the constraints that limit men's and women's productivity. When these new quantitative data are combined with rigorous qualitative analyses, they may provide guidance on how to best develop projects to ensure that women benefit.

The new data and the resulting analyses do not, however, answer all questions. In particular, they do not tell us very directly how to target interventions. If women provide 40% of the labour in crop agriculture, what does this fact tell us about whether (and how) to target interventions to women? Should we follow a simple rule of thumb and provide 40% of the resources to women and 60% to men? Should we ignore women altogether since their labour input is less than that of men? Either approach would seem to ignore the more important question—namely, where can we expect to find the highest rates of return and ensure that men and women both benefit.

Arguably, some of the narrow analyses of men's and women's productivity in agriculture have taken us away from the bigger and more important questions. The search for more precise estimates has spawned a rich and challenging literature, but it has diverged from several of the most important policy questions.

Can we target women for agricultural development interventions? If so, how? There are a number of promising approaches for targeting women in the agricultural sector.^7^ Some interventions are targeted specifically at women, while others seek ways to ensure women's participation in projects that do not have a gender focus. Key interventions for increasing women's agricultural productivity might not lie in the agricultural sector at all. Interventions that reduce women's drudgery, such as providing tap water or reducing the daily trek for firewood, could free up time and energy which could be more productively used in farming. Improving child health and nutrition could also reduce the care burdens that women face. But these interventions are hardly ever evaluated in terms of their impact on women's agricultural productivity.

In addition, this focus on comparing men's and women's agricultural productivity may lead us away from asking how interventions in the agriculture sector that include women may have impacts not only on agricultural output, but also on reducing poverty and increasing gender equity.

Whether or not interventions should explicitly *target* women rather than men, it is clear that a *gender‐blind* approach to designing interventions will miss out on key constraints, opportunities and impacts. Gender is embedded in the distribution of essentially all the resources used in agriculture—including land ownership, farm management decisions, market access for inputs and outputs, information from extension services, use of information and communication technology, etc. Gender is also embedded in the distribution of the gains from increased agricultural productivity, particularly influencing who controls the outputs and decides how the proceeds will be used. If interventions fail to consider how gender is embedded in the system, we will miss critical opportunities for transforming agricultural systems, increasing productivity, reducing poverty and improving people's lives.

## NOTES

1 Thus, the literature comparing male‐ and female‐headed households will not be included in the empirical review in this article. In spite of the problems of this comparison, it has been pervasively used in the literature, and researchers continue to characterize this as a form of gender analysis.

2 Accurately measuring land area is an additional challenge (see Carletto, Gourlay, Murray, & Zezza, 2016).

3 The LSMS‐ISA project is led by the World Bank and funded by the Bill and Melinda Gates Foundation. More information is available at http://go/worldbank.org/BCLXW38HY0

4 To calculate the profitability of particular crops requires information of the fertilizer used on the particular crop level on each plot. Again, this is challenging when intercropping is practiced; e.g. how should the cost of the input be allocated across crops?

5 See Doss (2007), for a much more detailed discussion of the technology adoption literature.

6 In an analysis of a dairy development project in Mozambique, Johnson et al., (2015) find that training two people within the household, instead of only the male household head, resulted in higher levels of milk production.

7 See Quisumbing and Pandolfelli (2010) and Doss et al., (2012) for reviews of such projects.
